# Recent advances in understanding and improving photosynthesis

**DOI:** 10.12703/b/9-5

**Published:** 2020-11-06

**Authors:** Alicia V Perera-Castro, Jaume Flexas

**Affiliations:** 1 Department of Biology, Universitat de les Illes Balears, INAGEA, Palma de Mallorca, Spain

**Keywords:** light harvesting, photosystem, rubisco, stomatal conductance, mesophyll conductance, engineering photosynthesis

## Abstract

Since 1893, when the word “photosynthesis” was first coined by Charles Reid Barnes and Conway MacMillan, our understanding of the elements and regulation of this complex process is far from being entirely understood. We aim to review the most relevant advances in photosynthesis research from the last few years and to provide a perspective on the forthcoming research in this field. Recent discoveries related to light sensing, harvesting, and dissipation; kinetics of CO_2_ fixation; components and regulators of CO_2_ diffusion through stomata and mesophyll; and genetic engineering for improving photosynthetic and production capacities of crops are addressed.

## Introduction

Photosynthesis is the chemical reaction that sustains most life on Earth. Since the description of the Hill reaction and the Calvin-Benson cycle^1–3^, knowledge about their components, regulation, and limitations experienced a vertiginous increase. It is widely known that plants have important handicaps related to photosynthesis. First, the photosynthetic apparatus that harvests and transforms light energy into electron transport for the generation of ATP and NADPH_2_ must cope with the generation of dangerous reactive oxygen species (ROS)^4^ and most of the energy must be “wasted” in dynamic heat dissipation mechanisms^5^. Second, the enzyme that catalyzes CO_2_ fixation in the Calvin-Benson cycle—ribulose 1,5-bisphosphate carboxylase oxidase or rubisco—is inefficient owing to several intrinsic characteristics, the most notable being the competitiveness between carboxylation and oxidation processes, since the oxidation of D-ribulose-1,5-bisphosphate results in the energetically expensive but perhaps convenient photorespiratory pathway^6^. And, third, the diffusion of CO_2_ from the atmosphere surrounding leaves through stomata and the leaf tissues to the carboxylation sites in the chloroplast stroma, where rubisco is located, is a dynamic pathway that is full of barriers and includes gaseous, lepidic, and aqueous phases, the latter with a small solubility and diffusivity for CO_2_.

In the last few years, researchers have tried to determine the limitations and components of the processes described above. Engineering photosynthesis targeting different aspects of photosynthesis and its regulation has also advanced. The aim of this review is to compile and organize these advances in photosynthesis from the last few years and suggest a next horizon for plant physiologists, ecologists, and geneticists.

## Light harvesting and use

Light energy is absorbed and transferred to the photosystem II (PSII) core by the light-harvesting complex II (LHCII). The way this absorption is regulated is relevant, since excessive and/or unbalanced exposure to light can lead to the generation of ROS and, in the long term, to the initiation of senescence processes^7^. Some isoforms of LHCII upregulate its transcription and translation as a response to high irradiance^8,9^, and their interaction with PsbS—a protein that plays a special role in photoprotection—has been described in detail^10^. Furthermore, Janil *et al*.^11^ discussed the enhanced dimerization of LHCII under strong light conditions as a photoprotective response partially responsible for the dissipation of excess excitation. In line with this, Albanese *et al*.^12^ recently described how the organization of PSII–LHCII supercomplexes changed with the diversification of land plants, contributing to their adaptability to different light environments. However, photoprotective processes and their ecophysiological implications remain far from fully characterized^5^. At the extreme opposite to excess light, shaded leaves within the canopy exhibit lower photosynthesis rates and slower activation of rubisco, stomata opening, and relaxation of photoprotection states. These delays, especially in rubisco activation, have been estimated to decrease wheat assimilation by 21% in shade to sun transitions^13^. Indeed, the fact that light is often in excess in the most illuminated leaves while limited in the shaded leaves within the canopy has led to the suggestion that lowering chlorophyll content may result not only in negligible effects on leaf-level photosynthesis rates but also in a higher distribution of light harvesting through the canopy, hence potentially enhancing whole plant photosynthesis rates and yield^14,15^. On the other hand, alterations of the canopy structure have also been suggested as a mechanism to improve light interception and canopy assimilation (see the recent review by Morales *et al*.^16^ and references therein), mainly through long-term breeding but also through hormonal and/or genetic means^17^.

Besides studies on the photosynthetic management of light *amount*, the effect of light *quality* on photosynthesis-related issues has also been addressed. It is widely known that growing under blue light conditions induces lower photosynthetic rates, increases the synthesis of carotenoids and anthocyanins and the photoprotection capacity, and decreases stomata size while increasing their density^18^. Light quality also affects the level of ROS and the expression of antioxidant enzymes^19^. Recently, Górecka *et al*.^20^ demonstrated that PsbS is not only a compulsory protein for enhancing dissipation of the excess of light energy as heat but also relevant for the red/blue light-associated enhancement of tolerance to UV-C and chloroplast signaling for light memory. A recent study has also described a species-specific response of photosynthesis to the quality of light independent of its intensity^21^. These interspecific differences in light response represent an opportunity to deeply understand the elements of light harvesting and their adaptation to different light environments.

## Rubisco kinetics and CO_2_-concentrating mechanisms

Interspecific variation of rubisco kinetics has also been a focus over the last several years. In two almost simultaneously published works, Hermida-Carrera *et al*.^22^ and Orr *et al*.^23^ assessed rubisco kinetics, their temperature dependency, and the aminoacidic replacements in the large subunit of rubisco in many crop species. Orr *et al*.^23^ extended their study to include 75 angiosperm species and found that some undomesticated plants presented inherently better rubisco kinetics, being thus a potential source for crop photosynthesis improvement. Iñiguez *et al*.^24^ and Flamholz *et al*.^25^ extended the analysis of differences in rubisco catalysis across the phylogeny and correlated them with the incidence of CO_2_ concentration mechanisms (CCMs), showing that organisms that had evolved CCMs tended to have faster rubiscos yet with lower affinity and specificity for CO_2_. Hermida-Carrera *et al*.^26^ found similar results when comparing rubisco catalytic traits of orchids and bromeliads with and without CCMs. These results suggest that equipping C_3_ crops with CCMs could be another strategy for fueling their photosynthetic capacity.

C_4_ photosynthesis is often envisaged as an efficient CCM and thus converting typical C_3_ crops into C_4_ has been a long-standing goal, resulting in the development of large-scale projects like the ongoing C4Rice (https://c4rice.com/), yet the goal has not been fully accomplished yet^27^. Furthermore, transitioning from mostly C_3_ to mostly C_4_ crops may be an efficient way to enhance productivity in a world exhibiting increased global aridity^28,29^, as it has been shown that in some cases C_4_ plants performed better under drought than did C_3_ species^30^. In the same vein, introducing crassulacean acid metabolism (CAM) into C_3_ crops has been suggested as a strategy to increase water use efficiency, i.e. to maximize CO_2_ fixation with minimum water loss through transpiration^31,32^. On the other hand, other CCMs like those found in algae and other aquatic organisms (e.g. pyrenoids and carboxysomes) have been reported to concentrate more CO_2_ around rubisco than C_4_ photosynthesis. Hence, while the C_4_ mechanism allows CO_2_ concentrations around rubisco of at least 10-times higher than those of the surrounding atmosphere^33^, eukaryotic algae like *Chlamydomonas* containing pyrenoids can concentrate CO_2_ 40-times^34^ and prokaryotic cyanobacteria possessing carboxysomes 100-times^35^ higher than the surrounding atmosphere. Consequently, the potential expression of cyanobacterial and algal CCMs in crop plants has been proposed as an opportunity to improve their photosynthesis^36^.

Despite the inefficiencies of light harvesting and rubisco, photochemical and/or biochemical limitations to photosynthesis are not larger than the diffusional limitations related to both stomatal and mesophyll resistances to CO_2_ in most of the studied species^37–45^. Gago *et al*.^46^ recently presented a compilation of photosynthetic limitations across land plants’ phylogenies, in which angiosperms showed a well-balanced distribution among biochemical, stomatal, and mesophyll limitations; photosynthesis in gymnosperms and ferns was co-limited mostly by stomatal and mesophyll limitations; and in bryophytes and lycophytes the mesophyll limitation largely predominated.

## Mesophyll conductance components

Mesophyll conductance to CO_2_ (*g*_m_) depends on several leaf structures that comprise the pathway from sub-stomatal cavities to carboxylation sites of rubisco. Intercellular air spaces, cell walls, plasma membranes, cytosol, double chloroplast membranes, and stroma offer resistance to CO_2_ diffusion. Values of *g*_m_ vary strongly among species, and short-term changes in *g*_m_ have been reported in response to many different environmental variables^46–49^, although a part of them could reflect methodological errors or uncertainties^50–52^. While interspecific differences are largely explained by anatomical traits^37–39,53,54^, short-term changes cannot be explained either by variable leaf anatomy or by the temperature coefficient reported for CO_2_ diffusion^55–57^. Consequently, it has been suggested that a biochemically facilitated CO_2_ diffusion must contribute to *g*_m_ instead of solely physical diffusion^56,58–60^. Short-term chloroplast movement, aquaporins, and carbonic anhydrases have been indicated as candidates^53,56,61^, although their actual involvement is far away from being conclusive.

For instance, despite the fact that chloroplast surface area facing intercellular airspaces per unit leaf area (*S*_c_/*S*) is one of the anatomical parameters more correlated with *g*_m_^37,53,54^, no evidence for an association between short-term changes of *g*_m_ and chloroplast movement or leaf anatomy has been found^57,62,63^, with the exception of *Arabidopsis* mutants with phytochrome-mediated impairment of the chloroplast avoidance response^64^. In a similar way, the contribution of carbonic anhydrases to *g*_m_ variations remains elusive and is a matter of ongoing debate^65^. The most recent studies showed that latitudinal variation of *g*_m_ correlates with variations in carbonic anhydrase activity^66,67^ and that a coupled inhibition of both *g*_m_ and carbonic anhydrases is obtained with treatment with mercuric chloride^68^. Han *et al*.^69^ also reported a decrease in the expression of carbonic anhydrase (*CA1*) during drought. On the contrary, Kolbe and Cousins^70^ did not find any variation in *g*_m_ in five lines of maize despite their differences in carbonic anhydrase activity.

The role of aquaporins as enhancers of CO_2_ diffusion across membranes has been widely reported^48,71^. Changes in *g*_m_ had been induced by inhibitors of aquaporins^68,72^ in transgenics^73–76^ and in mutants^77–80^. Direct measurement of the CO_2_ permeability of chloroplasts also revealed a 50% reduction in chloroplasts of an *Arabidopsis* aquaporin mutant as compared to the wild-type^81^. Despite these findings, Kromdijk *et al*.^82^ recently reported null differences in *g*_m_ among several knockout aquaporin mutants and wild-type, probably due to functional redundancy of aquaporin isoforms.

Additionally, the relative importance of these biochemical processes and anatomical traits in regulating *g*_m_ remains unknown. Furthermore, recent studies showed uncertainty about estimating some relevant anatomical parameters from microscopic images of 2D cross-sections compared to 3D microscopy, especially the mesophyll surface area exposed to air-filled spaces^83^ and chloroplast volume^84^. This could partially explain the differences in the *g*_m_ calculated from chlorophyll fluorescence and/or gas exchange and *g*_m_ calculated so far from anatomical models^38,39,53,85,86^. Earles *et al*.^87^ have emphasized the need to improve 3D techniques and models to properly characterize leaf-level photosynthesis in its whole complexity.

Within the anatomical components, *S*_c_/*S* and cell wall thickness (*T*_cw_) have been recognized as especially determinant for *g*_m_^46^. Besides the effect of *T*_cw_, an effect of cell wall composition and porosity in short- and long-term variations of *g*_m_ has been suggested^88,89^, and recently the first empirical evidence was provided. Thus, a reduction of *g*_m_ was observed by Ellsworth *et al*.^90^ in mutants with disrupted β-glucosyl polysaccharides of the cell wall. More recent studies have shown that the decrease of *g*_m_ provoked by drought, salinity, and low temperatures is coupled with variations in the relative levels of cellulose, hemicelluloses, and pectins^91,92^. More evidence is needed to understand how cell wall composition affects porosity and CO_2_ diffusion.

## Stomatal conductance

As mentioned above, an additional important limiting factor of photosynthesis is the stomatal conductance (*g*_s_). Several internal and environmental factors are widely known to affect *g*_s_. Stomatal shape, size, density, and clustering influence *g*_s_ and therefore photosynthesis^93^. These traits are established during leaf development and regulated by several phytohormones, especially abscisic acid (ABA)^94^. Light, CO_2_, and water supply also affect *g*_s_^95,96^.

The speed of *g*_s_ responses to light and CO_2_ has been recently compared among phylogenetic plant groups. Although fern and lycophyte stomata are not insensitive to light and CO_2_, their response is lower and slower than that observed in angiosperms^97–100^. Furthermore, unlike angiosperms, fern and lycophyte stomata do not respond to endogenous levels of ABA^97,98^ and their closure is based on a passive response of guard cells to dehydration^101^. The mechanism that explains this different response remains unclear, although it is likely related to differences in the molecular mechanisms operating in the guard cells along the phylogeny. Among other factors affecting *g*_s_ (kinases, anion channels, etc.), it is known that carbonic anhydrases can be involved in the biochemical mechanism by which guard cells of angiosperms sense CO_2_ (see the review by Engineer *et al*.^95^), although details of signal transduction and the identity of the second messengers (bicarbonate, protons) are still debated. Furthermore, a higher CO_2_ assimilation related to phosphoenolpyruvate carboxylase activity followed by gluconeogenesis and maybe sucrose synthesis has been described for guard cells in comparison to those of mesophyll cells of C_3_ plants^102^.

In addition, recent studies suggest that stomata movement is regulated by mesophyll-derived signals. Sucrose has been identified as an important metabolite for the regulation of stomatal opening and closure^100,103,104^. Wang *et al*.^105^ reported that the maize mutant *cst1*—with an impaired membrane glucose transporter CST1 located in the subsidiary cell membrane—presented lower *g*_s_, lower photosynthesis, and earlier senescence than the wild-type. In line with this, Fujita *et al*.^106^ demonstrated that stomatal responses are disrupted when a membrane excluding molecules of 100–500 Da is transplanted between mesophyll and guard cells, which would avoid the transport of sucrose, malate, and ABA. In a study of ABA-regulated genes in *Arabidopsis*, Yoshida *et al*.^107^ found highly expressed genes in guard cells related to the tricarboxylic acid cycle and sucrose and hexose transport and metabolism. These studies support the hypothesis of stomatal regulation driven by carbohydrate/hormone-related mesophyll signals. However, the differences in the mechanism of mesophyll cell signaling and in guard cell metabolism among fern, lycophytes, and angiosperms—both anisohydric and isohydric species—remain unknown.

Even in angiosperms, the predominance of hormonal *vs*. hydraulic stomatal regulation is currently under debate^108–110^. Traditionally, stomatal closure has been understood as a safety valve to prevent cavitation (see Hochberg *et al*.^111^ and references therein). However, a detailed chronological description of the drought response of *g*_s_ and hydraulic conductance (*K*_leaf_) in rice revealed that the decline in *K*_leaf_ preceded and probably triggered the decline of *g*_s_ and *g*_m_^108^. Nadal *et al*.^112^ suggested that both types of drought response are not necessarily incompatible and can be related to the spectrum of the iso-anisohydric response of angiosperms.

## Engineering photosynthesis

While there are some opposing views^113^, improving photosynthesis is often envisaged as an important goal for improving crop yields^114–117^, including the cultivation of photosynthetic microorganisms, which constitutes a huge and important branch of bioengineering for bioenergy production^118,119^. Regarding land plant bioengineering, optimizing production with a minimum investment of resources (water, land, and nutrients) is the aim of ongoing large-scale projects, such as the already mentioned C4rice or the RIPE project (https://ripe.illinois.edu/). Several targets for manipulation—including all those mentioned in the above sections—have been proposed with the aim of improving photosynthesis and crop yield^120,121^. Neglecting which are the main limitations for photosynthesis when targeting genes for improving photosynthesis is an example of the mutual disregard that ecophysiologists and biotechnologists have had for each other in the last few decades^122^, i.e. biotechnologists attempting to improve photosynthetic targets that ecophysiologists were showing to be non-limiting for photosynthesis. Using a model approach, Flexas^116^ showed that only modest improvements of photosynthesis can be expected from relaxing only one limiting factor, since photosynthetic limitations are generally well-balanced in angiosperms^46^. Nevertheless, even with this relatively modest approach, increases of yield of >40% have been reported in some successful attempts^117^.

Rubisco kinetics have been among the most common targets for improving photosynthesis. All the advances in rubisco engineering have implied important improvements in our understanding of rubisco regulation and assembly but unsuccessfully improved the catalytic performance of rubisco^123,124^ or photosynthesis^125^. While faster rubisco from cyanobacteria have been successfully engineered in transplastomic tobacco^126^, post-transcriptional assembly of functional rubisco in large enough quantities remains a limiting factor, likely due to the inability of local chaperones to deal with foreign rubisco fragments (see Whitney *et al*.^127^ and their attempt to solve this problem by the use of ancillary chaperone genes). For this reason, this is a very active area of ongoing research^127,128^. Rubisco activase is another potential limiting factor, as Fukuyama *et al*.^129^ also showed how increased expression of rubisco activase resulted in a negative correlation with rubisco content.

Besides achieving more efficient rubiscos, an alternative strategy has been to increase CO_2_ concentration by either introducing elements of algal CCMs or bypassing photorespiration by different processes. While theoretically CCMs should increase photosynthesis^130^, introducing CCMs into either tobacco^131^ or *Arabidopsis* failed to increase photosynthesis^132,133^, probably because of insufficient encapsulation of local rubisco in the foreign carboxysomes, which can be improved by simultaneously replacing the native large subunit of rubisco^134^. Additional elements might also be essential for a proper assemblage of fully functional carboxysome–rubisco CCMs, as recently demonstrated for bestrophin-like proteins^135^.

More successful results have been obtained when the photorespiration pathway has been manipulated in *Arabidopsis* and tobacco^136,137^. While photosynthesis increases^136^, biomass production has been shown to vary from decreasing through unaffected to increasing by 10–50%^117,138^. Recently, South *et al*.^137^ obtained a 24% maximum increase of biomass when glycolate byproducts of photorespiration are processed by foreign malate synthase and a green algal glycolate dehydrogenase, substituting the native pathway. Tissue-specific overexpression of one of the subunits forming in the glycine dehydrogenase system also increased biomass yield by 13–38% in tobacco^139^. This is a very promising approach for improving grain crop yields in the near future.

Also, modifications of the Calvin-Benson cycle have resulted in improved photosynthesis and yield. Overexpression or transgenic insertion of several enzymes involved in the cycle (mostly sucrose bis-phosphatase—SBPase—and fructose bis-phosphatase—FBPase—but also FBPaldolase) has also resulted in increased photosynthesis and dry weights, although generally not in improved yield. However, Driever *et al*.^140^ showed an up to 40% increase in grain yield in wheat, and Simkin *et al*.^141^ a 35–53% increase in seed yield in *Arabidopsis*. Furthermore, overexpression of FBP/SBPase has been recently combined with an improved electron transport by the addition of the algae cytochrome C_6_, which also resulted in up to 53% of increase of biomass^142^. These results open up the possibility of using this approach for improving crop yields in the very near future.

Few attempts have focused on modifying CO_2_ diffusive characteristics of leaves. Altered stomatal density in epidermal patterning factor (EPF) mutants of *Arabidopsis*^143^ and wheat^144^ resulted in an increased photosynthetic water-use efficiency (WUE) but not increased photosynthesis itself. Similarly, Yang *et al*.^145^ showed that overexpression of the ABA receptors RCAR6/PYL12 increases the sensitivity of the stomata in *Arabidopsis* lines, reducing g_s_ even in the absence of water stress without affecting photosynthesis, thus also enhancing WUE. As described in previous sections, *g*_s_ was also enhanced by overexpression of glucose transporters in subsidiary cell membranes^105^.

Generally speaking, increasing stomatal conductance does not result in enhanced photosynthesis because stomatal limitations are generally minor in the absence of stress. However, during leaf development, the presence of well-developed and functional stomata appears to be the main driver of the development of mesophyll porosity, which is an essential anatomical trade favoring *g*_m_ and hence photosynthesis^146^. This finding is remarkable as it implies that, while it is likely that a mesophyll signal is involved in stomata regulation (see above sections), stomata define the developmental set-up of the mesophyll structure, hence establishing a very intricate co-dependency between *g*_s_ and *g*_m_ limitations at different time scales that deserves further study. In line with this, Lehmeier *et al*.^147^ showed that it is possible to genetically modify cell density and the arrangement of the air channels with an overall decreased path tortuosity in the palisade air spaces in a way that facilitates *g*_m_ without affecting *g*_s_. Similarly, alteration of leaf mesophyll anatomy of *Eucalyptus* has been attempted by the overexpression of the transcription factor *EcHB1*, which is involved in multiple genes related to cell wall biosynthesis and cell growth, increasing the number of chloroplasts per unit leaf area and therefore enhancing CO_2_ diffusion into chloroplasts and photosynthesis^148^. These results offer new possibilities in improving photosynthesis by reducing CO_2_ diffusion limitations. Advances in the understanding of cell wall composition determinants of *g*_m_ may open complementary doors in the near future.

While significant and important in some cases, the above-described manipulations aimed to improve maximum photosynthesis rates, i.e. light-saturated photosynthesis in the absence of abiotic and biotic stresses. However, photosynthesis in nature occurs in largely variable conditions, e.g. in fluctuating light. For instance, De Souza *et al*.^43^ showed in cassava that, while under steady-state high-light conditions, *g*_m_ and biochemical limitations accounted for up to 84% of the total photosynthetic limitation and, under non-steady state conditions during shade to sun transition, *g*_s_ became the most dominant limitation. Thus, in recent years, research has focused on improving photosynthesis and efficiency under non-steady-state conditions by decreasing the excess absorption of light^15,149^ or increasing the relaxing velocity of photoprotection^150–152^. More surprisingly, overexpressing PsbS in transgenic tobacco resulted in enhanced WUE by reducing *g*_s_, not increasing photosynthesis, again pointing to potential mesophyll signals in stomata regulation^153^. Recently, Papanatsiou *et al*.^154^ used an optogenetic approach to improve photosynthesis, WUE, and growth in *Arabidopsis*. They expressed a synthetic light-gated K^+^ channel in stomatal guard cells (BLINK1), which improved the speed of stomata kinetics in response to varying light. Increased velocity of stomata opening from a dark-to-light transition and closing from a light-to-dark transition resulted in increased plant growth and WUE by approximately 30%^154^.

## Conclusion

Light sensing, photoprotection, CO_2_ diffusion, and its fixation involve numerous and complex processes that are far from fully understood. In the last few years, new insights have been obtained into how interaction and conformation of light-harvesting complexes and photosystems affect photoprotection and heat dissipation. Advances have been made also in the understanding of the variability in rubisco kinetics and photosynthetic limitations at steady state along the plant’s phylogeny, of the genetics and mechanistic aspects of carbon-concentrating mechanisms, and of the major anatomical determinants of *g*_m_ and the metabolic determinants of stomatal conductance and kinetics. Important links between mesophyll and stomatal cells have been revealed, although the signaling between mesophyll cells and guard cells that regulates *g*_s_ requires further research, as does understanding the chemical and biochemical determinants of *g*_m_.

Nevertheless, owing to the new knowledge acquired, engineering efforts for improving photosynthesis and photosynthetic WUE have been attempted, some of them with significant success, which open up the opportunity for photosynthesis-mediated improvement of crop productivity in the forthcoming years. To achieve this goal, a close collaboration among plant physiologists, molecular biologists, geneticists, and agronomists might be essential for generating multiple new photosynthetic genotypes and evaluating them under realistic conditions, both under steady- and non-steady-state conditions, from a photosynthetic limitations perspective to a yield and WUE perspective^122^. Technical advances in analytical tools, like the recently implemented rapid CO_2_ response curves of gas exchange^155–159^, would be crucial to allow in-depth phenotyping of photosynthesis in record times.
